# Deficiency of αII-spectrin affects endothelial cell–matrix contact and migration leading to impairment of angiogenesis in vitro

**DOI:** 10.1186/s11658-020-0200-y

**Published:** 2020-02-03

**Authors:** Beata Machnicka, Aurélie Ponceau, Julien Picot, Yves Colin, Marie-Christine Lecomte

**Affiliations:** 1 University of Zielona Góra, Institute of Biological Sciences, Zielona Góra, Poland; 2 Biologie Intégrée du Globule Rouge, UMR_S1134, BIGR, INSERM, Université de Paris, F-75015 Paris, France; 3 Institut National de la Transfusion Sanguine, F-75015 Paris, France; 4 Laboratoire d’Excellence GR-Ex, Paris, France

**Keywords:** αII-spectrin, Endothelial cells, Adhesion, Migration, Actin cytoskeleton, Capillary tube assembly

## Abstract

**Background:**

Precise coordination of cytoskeletal components and dynamic control of cell adhesion and migration are required for crucial cell processes such as differentiation and morphogenesis. We investigated the potential involvement of αII-spectrin, a ubiquitous scaffolding element of the membrane skeleton, in the adhesion and angiogenesis mechanism.

**Methods:**

The cell models were primary human umbilical vein endothelial cells (HUVECs) and a human dermal microvascular endothelial cell line (HMEC-1). After siRNA- and shRNA-mediated knockdown of αII-spectrin, we assessed its expression and that of its partners and adhesion proteins using western blotting. The phenotypes of the control and spectrin-depleted cells were examined using immunofluorescence and video microscopy. Capillary tube formation was assessed using the thick gel Matrigel matrix-based method and a microscope equipped with a thermostatic chamber and a Nikon Biostation System camera.

**Results:**

Knockdown of αII-spectrin leads to: modified cell shape; actin cytoskeleton organization with the presence of peripheral actin patches; and decreased formation of stress fibers. Spectrin deficiency affects cell adhesion on laminin and fibronectin and cell motility. This included modification of the localization of adhesion molecules, such as αVβ3- and α5-integrins, and organization of adhesion structures, such as focal points. Deficiency of αII-spectrin can also affect the complex mechanism of in vitro capillary tube formation, as demonstrated in a model of angiogenesis. Live imaging revealed that impairment of capillary tube assembly was mainly associated with a significant decrease in cell projection length and stability. αII-spectrin depletion is also associated with significantly decreased expression of three proteins involved in capillary tube formation and assembly: VE-cadherin, MCAM and β3-integrin.

**Conclusion:**

Our data confirm the role of αII-spectrin in the control of cell adhesion and spreading. Moreover, our findings further support the participation of αII-spectrin in capillary tube formation in vitro through control of adhesion molecules, such as integrins. This indicates a new function of αII-spectrin in angiogenesis.

## Introduction

The peripheral protein network of the red blood cell (RBC) membrane is mainly made of spectrin, actin and protein 4.1. Many studies on RBC, particularly analyses of mutations causing hereditary hemolytic anemia, have defined the importance of this network for maintaining cell shape and membrane integrity [1, 2].

The fundamental member of this network, spectrin, is thought to be present in all metazoan cells. In non-erythroid cells, it also occurs in endomembranes of the nucleus, cytoplasmic vesicles and the Golgi complexes [3]. Recent studies have shown that similar spectrin-based structures participate in both the establishment and maintenance of many highly specialized membrane domains that enable the cell to execute a variety of physiological functions, including morphogenesis [4–7].

In mammalian RBCs, spectrins mainly occur as large and flexible heterotetramers made of a set of two αI and two βI subunits. These tetramers, as basic filaments of the network, cross-link short actin filaments via the actin-binding domain present in β-spectrins. In nucleated cells, there are a large number of possible combinations of spectrin isoforms. They are expressed from two genes that code α-spectrins (αI and αII subunits) and five that code β-spectrins (βI through βV subunits) [8].

The occurrence of the spectrin-based skeleton in various cellular environments and its interaction with multiple proteins indicate that spectrin plays a role in many different physiological pathways involved in cell proliferation and differentiation. Some studies show that spectrin is essential for normal embryogenesis and organ development [9]. For example, αII-spectrin appears to be a key component in cell spreading, tissue control and organ development in vertebrates [10]. Furthermore, in *Drosophila*, cortical β(H)-spectrin is directly involved in the function of sosie, which an essential gene for oogenesis. Dysfunction of sosie in the germ and soma cell lines of *Drosophila* leads to defective organization of cytoplasmic actin networks [11].

Our recent studies also confirmed the contribution of αII-spectrin in cell adhesion processes and in organization of the actin cytoskeleton in various cell models. siRNA-mediated depletion of αII-spectrin in a melanoma cell line revealed defects in cell adhesion, such as changes in actin stress fibers, modification of focal adhesion and altered levels of some integrins [12]. Such alterations have also been observed in embryonic fibroblasts from αII-spectrin^−/−^ mice [10]. In human neuroblastoma cells, depletion of αII-spectrin causes loss of adhesive properties in cell bodies and neurites [13]. Moreover, spectrin may also regulate the function and development of actin-rich, dynamic invadosomes by controlling the mobility of the integrins in the membrane [14]. Furthermore, the regulatory role of spectrin in cell–cell contact and adhesion processes in the first stages of immunological synapse (IS) formation was recently demonstrated. Loss of αII-spectrin was associated with loss of actin-rich lamelipodias in activated T lymphocytes [15].

In this study, we used different endothelial cell (EC) models to investigate the involvement of αII-spectrin in: cell adhesion to the extracellular matrix; cell motility; and actin cytoskeleton dynamics. We further analyzed the impact of αII-spectrin depletion on the assembly of capillary tubes in vitro to ascertain its role in modulating endothelial migration during angiogenesis.

## Experimental procedure

### Cell culture

The human microvascular endothelial cell line HMEC-1 (ATCC, CRL-10636) was grown in MCDB131 (Gibco) supplemented with 15% FCS (FCS PAN Biotech), 2 mM L-glutamine, 1 μg of dexamethasone (D8893, Sigma) and 100 ng of EGF (Invitrogen). The human umbilical vein endothelial cells (HUVECs) were cultured in M199 containing 20% FCS, 1% L-glutamine and 10 mM HEPES. The culture media contained 1% penicillin (10^3^ units/ml) and streptomycin (10^3^ μg/ml) (Invitrogen). Cells were plated on plastic coated with gelatin 0.2% (Sigma) and incubated at 37 °C in a water-saturated atmosphere with 5% CO_2_. The HUVECs used for the experiments were from passages 1 to 5 and the HMEC-1 cells from passages 10 to 25.

### Transfection

HUVECs and HMEC-1 cells were transfected with either siRNA heteroduplexes or GFP-shRNA (Sp shRNA) plasmids using the HUVEC Nuclofector Kit (Amaxa Biosystem) and the general JET PEI transfection reagent (Polyplus), respectively, according to the manufacturer’s instructions. The culture medium was changed 24 h after transfection to remove transfection products.

Transfection efficiency was estimated via flow cytometry using a FACSCalibur flow cytometer (BD Biosciences) either with control siRNAs (non-relevant siRNA, Nr siRNA) labelled with Alexa Fluor 488 or 568 (Qiagen) for HUVEC and HMEC-1 cells, or with control plasmids expressing both non-relevant shRNA (Nr shRNA) and GFP for HMEC-1 cells. Cell viability was also determined using flow cytometry 24 h after transfection in the presence of 5 μg/ml propidium iodide (PI).

The siRNA duplexes targeting human αII-spectrin (Sp siRNA) were Dharmacon Individual siGENOME duplex D-009933-01, D-009933-02, D-009933-03 and D-009933-04, and Ambion Silencer Pre-designed siRNAs 12,798 and 142,727. The negative silencer control siRNAs (Nr siRNA) were Dharmacon siCONTROL Non-Targeting siRNA Pool. The shRNA plasmids targeting human αII-spectrin (Sp shRNA) were SABiosciences KH18852G clone 1, 2, 3 and 4. We validated the efficiency of Sp siRNA 1 and 17 and Sp shRNA 3 and 4 in our experiments.

### Antibodies

Monoclonal antibodies directed against vinculin were obtained from Sigma-Aldrich; αVβ3-integrin from Chemicon International; α5-integrin from BD Biosciences; and αII-spectrin clone AA6 from Biohit and Millipore. Polyclonal antibodies against lamin A/C were purchased from Santa Cruz Biotechnology; VASP and ABI-1 from Sigma-Aldrich; and VE-cadherin from Abcam. Polyclonal antibodies directed against αII-spectrin were obtained after the immunization of guinea pig (Eurogentec) using the recombinant peptide SH3-α8-α11-His_6_ αII-spectrin repeats.

### Western blot analysis

After two washes with pre-warmed Dulbecco’s PBS (Gibco), the cells were directly lysed on plates in PBS (137 mM NaCl, 2.7 mM KCl, 10 mM Na_2_HPO_4,_ 1.8 mM KH_2_PO_4_) containing 1% SDS, anti-protease cocktail (Sigma) and OmniCleave endonuclease (Epicentre). Protein concentrations were estimated in a colorimetric assay using the BCA method (microAssay Uptima) with BSA as the standard protein. Aliquots of cell lysates (between 20 and 40 μg) were resolved on SDS poly-acrylamide gels and transferred onto Protan nitrocellulose membrane (0.45 μ; Schleicher & Schuell) using a Tris-glycine buffer. After saturation in 5% non-fat-milk, 0.05% Tween 20, PBS buffer (pH 7.5), the membranes were probed overnight at 4 °C with the indicated primary antibodies. After extensive washing, blots were incubated for 1 h at room temperature with secondary antibodies conjugated with horseradish peroxidase (Nordic Immunological laboratories). Immune complexes were detected using the Supersignal West Pico chemiluminescence substrate (Pierce). The chemiluminescence was quantified using the Quantity One 1-D Analysis software (Bio-Rad) after acquisition with Molecular Imager Gel Doc (Bio-Rad).

### Immunofluorescence studies

Cells grown on CC2 or permanox slides (Nunc) were washed in pre-warmed Dulbecco’s PBS, fixed in 4% paraformaldehyde, permeabilized with 0.5% Triton X-100 and saturated for 30 min either with buffered saline solution containing 0.1% BSA or with Image-iT Signal Enhancer (Molecular Probes). Primary and secondary antibody dilutions were made in background-reducing buffer (DakoCytomation, Invitrogen). In a final step, immunolabelled cells were mounted in ProLong Antifade Gold solution. Secondary labelled anti-IgG antibodies were purchased from Molecular Probes (Alexa Fluor 488 and 568). F-actin was labelled with Alexa Fluor 568 or 488 phalloidin (Molecular Probes). The fluorescence was observed via confocal microscopy using either a Zeiss LSM 510 META or a Nikon Eclipse TE300 microscope upgraded with a D-Eclipse C1 confocal system.

### Static cell adhesion assays

Adhesion assays were performed 48 h after transfection on culture dishes coated with either fibronectin or laminin 510/511. Control and transfected cells were respectively stained with the vital dyes Hoechst 33342 (1 μg/ml) and calcein (10 μg/ml) AM (Molecular Probes). After washing in D-PBS, the cells were detached using trypsin-EDTA and washed in complete culture medium, then both labelled cell populations were mixed in a 1/1 ratio. These mixtures were plated in triplicate on 12-well plates (2 × 10^6^ cells per well) and incubated for 2 h at 37 °C in 5% CO_2_. After two washes with complete warmed culture medium, the remaining adherent cells were visualized via fluorescence using an Evolution VF camera (Media Cybernetics). Ten images were acquired for each sample of mixed cells. Adherent cells were counted using Image-Pro Plus software. The results are expressed as the mean percentages of adherent transfected cells versus adherent control cells (100%). Spread cells were discriminated from round cells based on the fluorescence intensity of Calcein and Hoechst: the round cells showed a higher intensity.

### Study of αII-spectrin distribution during the adhesion process

HMEC-1 cells were plated on CC2 slides coated with either fibronectin or laminin and incubated for 5, 10 and 30 min at 37 °C in 5% CO_2_. Cells were gently washed once with complete culture medium before fixation with 4% paraformaldehyde. The remaining adherent cells were detected using immunofluorescence after actin and αII-spectrin labelling.

### Study of capillary tube formation via video microscopy

Capillary tube formation was assessed using the thick gel Matrigel matrix-based method. Matrigel Basement Membrane Matrix Phenol Red Free (BD Biosciences) was first thawed at 4 °C for one night then mixed using cooled pipettes before being used to coat permanox coverslips on ice. Incubation was at 37 °C for 30 min to allow gel polymerization.

Dynamic studies of capillary tube formation were performed in HMEC-1 cells transfected with plasmids expressing GFP and either non-relevant shRNA (Nr shRNA-GFP) or shRNA targeted αII-spectrin (Sp shRNA-GFP). Cells were plated on permanox slides coated with Matrigel at a density of 10^5^ in a 2 ml final volume and then incubated for 12 h at 37 °C in 5% CO_2_ in the Biostation system. Capillary tube formation was followed using a microscope equipped with a thermostatic chamber and a camera (Biostation system, Nikon). Images were registered every 2 min. Three principal characteristics were measured: the mean number of filopodia per cell, their maximal size and their stability. From three different experiments (analysis of 10 images per experiment), the number of filopodia was calculated by counting the maximal number of filopodia or other extensions formed by cells. The size of the filopodia was measured when filopodia were at their maximum length in the same images. Their stability was assessed by measuring the time interval between the beginning of filopodia formation and their retraction.

### Statistical analysis

The statistical significance was mainly calculated using Student’s test. Capillary tube experiments were analyzed using two tests: the Bartlett test for comparison of variance and the Mann-Whitney test.

## Results

### Spectrin depletion modifies the actin cytoskeleton in endothelial cells

**Fig. 1:**
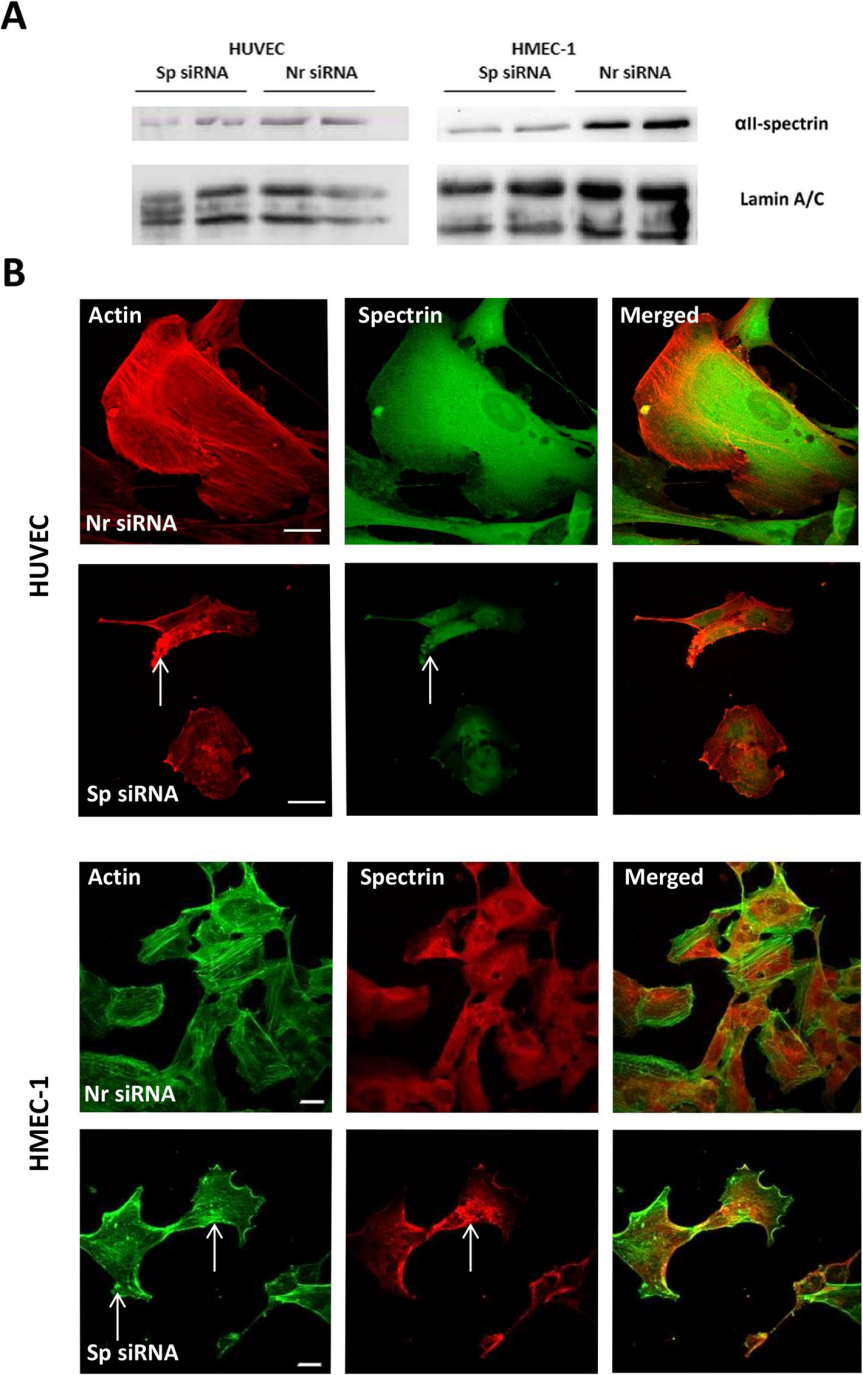
αII-spectrin depletion in endothelial cells modifies their shape and actin cytoskeleton. **a** Western blotting of αII-spectrin in HMEC-1 cells and HUVECs. Lysates (20 μg) of cells transfected with either siRNAs targeting αII-spectrin (Sp siRNA) or non-relevant siRNA (Nr siRNA) were analyzed 72 h after transfection. αII-spectrin and lamin A/C (used as a loading control) levels were checked using polyclonal antibodies. Knockdown of αII-spectrin was efficient with two siRNAs (see the Experimental procedure section). Residual expression of αII-spectrin in Sp siRNA-transfected cells was about 20 to 30%. The transfection efficiency in the cells was about 95% (as evaluated by flow cytometry, data not shown). **b** Analysis of cell morphology and actin cytoskeleton. HMEC-1 cells and HUVECs were transfected with siRNAs targeting either αII-spectrin (Sp siRNA) or non-relevant siRNA (Nr siRNA). Cells were labelled 72 h after transfection with polyclonal antibodies directed against αII-spectrin (labelled green in HUVECs and red in HMEC-1 cells) and phalloïdin toxin detecting actin (red in HUVECs and green in HMEC-1 cells). Sp siRNA transfection induces a decreased labelling of αII-spectrin that accumulates into some aggregates (arrows). Spectrin depletion also modifies the actin architecture in endothelial cells: depleted HUVECs and HMEC-1 cells present a marked phenotype with disorganization of stress fibers, patches and aggregates (arrows). Scale bar = 20 μm

We previously found that spectrin deficiency in a human melanoma cell line [12] and in T cells [15] is associated with modifications of the actin cytoskeleton and with defects in cell adhesion, spreading and cell–cell contact. To test whether these events, which are associated with spectrin loss, are common features in any cellular context, we here investigated the effects of spectrin knockdown using a RNAi strategy in two endothelial cell lines: HMEC-1 and HUVECs. Using at least two different siRNA or siRNA pools, a roughly 70% decrease in spectrin expression was obtained in both cell types as evaluated via western blot (Fig. 1a, Additional file 5: Figure S4).

Immunofluorescence showed that HUVEC and HMEC-1 cells treated with non-relevant siRNA (Fig. 1b) exhibit phenotype control cells (non-transfected cells, Additional file 1: Figure S1): the cell shape is not modified and the cells are well spread. Alpha II-spectrin labelled with different antibodies (mono- and polyclonal) is mainly present in the cytoplasm and around the nucleus, with weak labelling in the nucleus. Spectrin is also present at the cell membrane and at the leading edge, where it can accumulate as some patches (Additional file 1: Figure S1). In most cells, phalloidin labelled actin is present mainly as stress fibers.

In cells treated with siRNA targeting αII-spectrin, spectrin labelling is reduced, indicating efficient depletion (Fig. 1b). Decreased spectrin expression is associated with important modifications in cell shape, as manifested in the reduction in the size and number of adherent cells. In αII-spectrin-depleted cells, the remaining spectrin accumulates as aggregates in the cytoplasm. Phalloidin labelling showed disorganization of the actin network in the form of a reduction in the basal stress fibers and the presence of actin patches or aggregates.

Video microscopy analysis of cells transfected with both GFP-actin and siRNAs (Nr and Sp) confirmed the observations obtained on fixed cells (Additional file 2: Figure S2): spectrin-depleted cells had reduced size with disorganization of the actin network and loss of stress fibers. In cells transfected with a non-relevant siRNA, GFP-actin produces very dynamic stress fibers and accumulates at the leading edge. By contrast, in spectrin-depleted cells stress fibers are not formed and GFP-actin accumulates in dynamic patches. These modifications within the actin skeleton, mainly the disappearance of stress fibers, indicate the links between spectrin and the actin-based cytoskeleton.

### Cell adhesion and spreading are impaired in spectrin-deficient cells

The smaller size of spectrin-depleted cells correlates with impaired spreading. This raises the question of potential associated defects in cell adhesion and spreading. For endothelial HMEC-1 cells, these processes were assessed based on two cell matrix components: fibronectin and laminin 510/511.

**Fig. 2:**
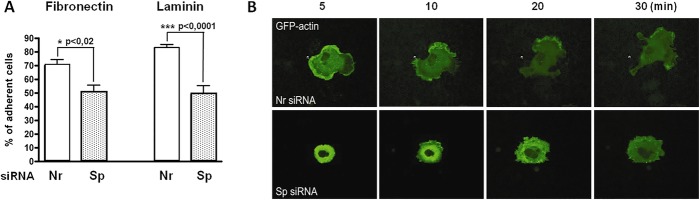
Knockdown of αII-spectrin is associated with defective cell adhesion and spreading. **a** Static cell adhesion assay: 72 h after transfection with either non-relevant siRNA (Nr siRNA) or siRNA targeting αII-spectrin (Sp siRNA), HMEC-1 cells were tested for their ability to bind on fibronectin and laminin. The percentage of adherent Sp siRNA-treated cells 2 h after plating is significantly lower than that for control or Nr siRNA-treated cells on both substrates **b** Kinetic analysis of adhesion of primary HUVECs on laminin. HUVECs expressing GFP-actin were transfected with either Sp siRNA or Nr siRNA. Adhesion on laminin was followed using video microscopy

Static adhesion assays performed 72 h after siRNA transfection showed a statistically significant decrease in the number of adherent cells treated with siRNA targeting spectrin compared to the number of adherent cells treated with non-relevant siRNA (as described in the Experimental procedure section). Compared to control cells, the percentage of remaining adherent cells treated with Sp siRNAs (obtained in four independent experiments) was around 50% on both matrices (Fig. 2a), while the number of adherent cells treated with Nr siRNA was reduced by 10 to 30% based on the matrix component.

The analysis of HMEC-1 cells 2 h after seeding on fibronectin, revealed a defective spreading for cells treated with siRNAs targeting spectrin. The ratio of spread versus non-spread cells in non-relevant siRNA-treated samples was 2.0, whereas it was three times less (0.7) in spectrin-depleted cells.

The beginning of the spreading process was followed in living cells transfected with both GFP-actin and siRNAs. At the beginning of the adhesion process (Fig. 2b, 5 min) and during spreading (Fig. 2b, 10 to 20 min), Nr siRNA-treated cells spread well, were motile and presented highly dynamic actin-rich lamellipodia (Fig. 2b and Additional file 3: Figure S3A and Additional file 4: Figure S3B). In spectrin-depleted cells, lamellipodia were less dynamic and actin was present in patches, confirming previous observations of fixed cells. The cells were smaller and more rounded. They also exhibited a lower mobility. All the Nr siRNA-treated cells were completely spread at 30 min after plating, whereas the spectrin-depleted cells remained rounded (Fig. 2b, 30 min). Therefore, spectrin depletion induces a defect in adhesion and spreading in both endothelial cell types.

### Spectrin is recruited at the leading edge during adhesion and spreading

**Fig. 3:**
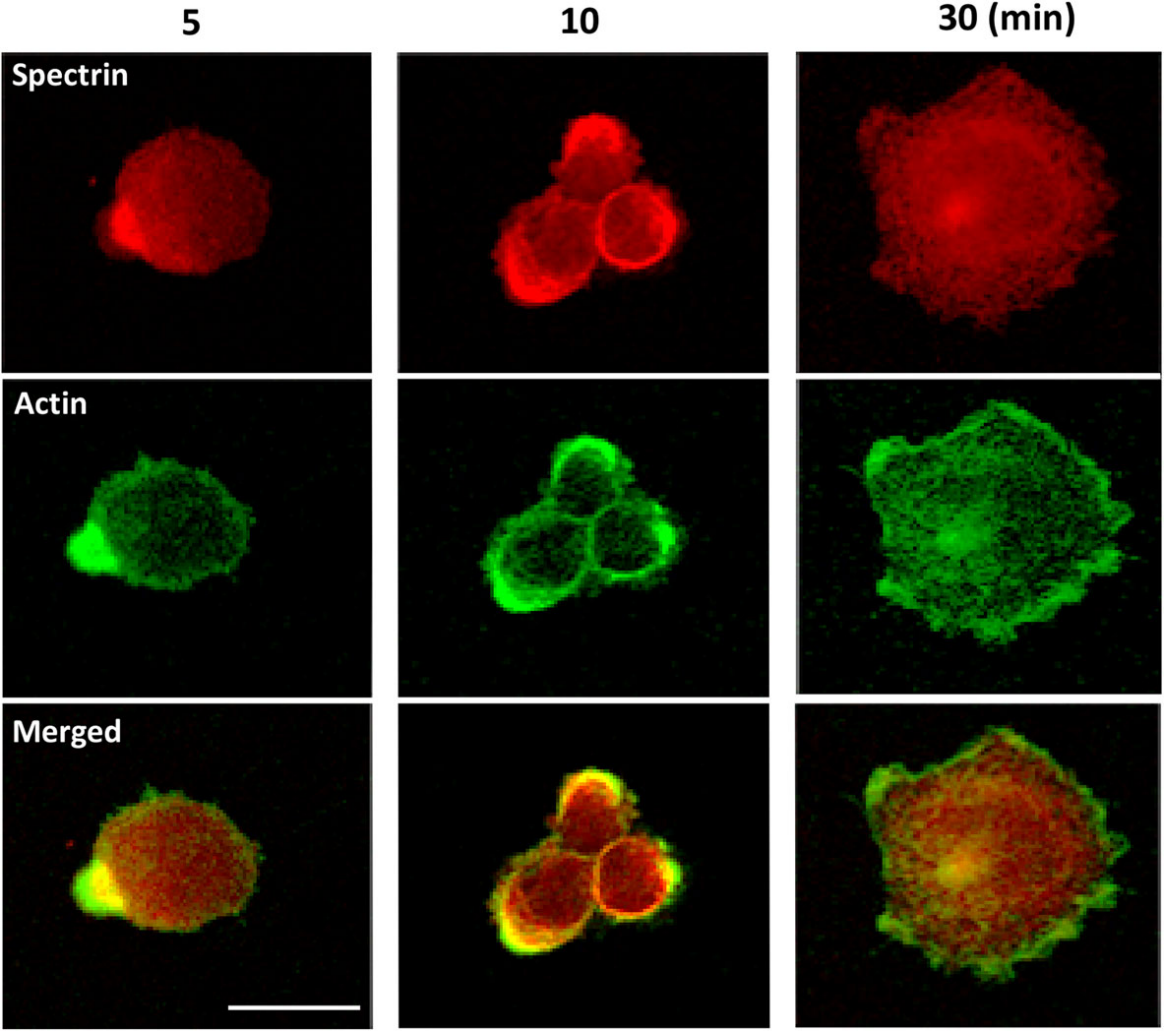
αII-spectin is present in cell budding during adhesion. HMEC-1 cells were fixed at 5, 10 and 30 min after seeding on fibronectin. αII-spectrin was labeled with polyclonal antibody (red) and actin with phalloïdin toxin (green). Scale bar = 10 μm

As αII-spectrin loss in endothelial cells causes a significant defect in cell adhesion and spreading, the localization of αII-spectrin during these processes was investigated via immunofluorescence. HMEC-1 cells were plated on fibronectin-coated slides and fixed at different times. At the beginning of the adhesion process (5 and 10 min), αII-spectrin accumulates in the cell budding and is partially colocalized with actin at their bases, with the actin more at the front of the membrane (Fig. 3). This particular accumulation of spectrin was observed not only in human endothelial cells, but also in human WM266 and Jurkat cells and mouse BF16 melanoma cells (data not shown). At 30 min after plating, the cells are well spread, and actin is mainly present at the front of the membrane Spectrin labelling is mainly found in the cytoplasm with weak labelling at the membrane.

### Adhesion structures such as focal points are modified in spectrin-depleted cells

**Fig. 4:**
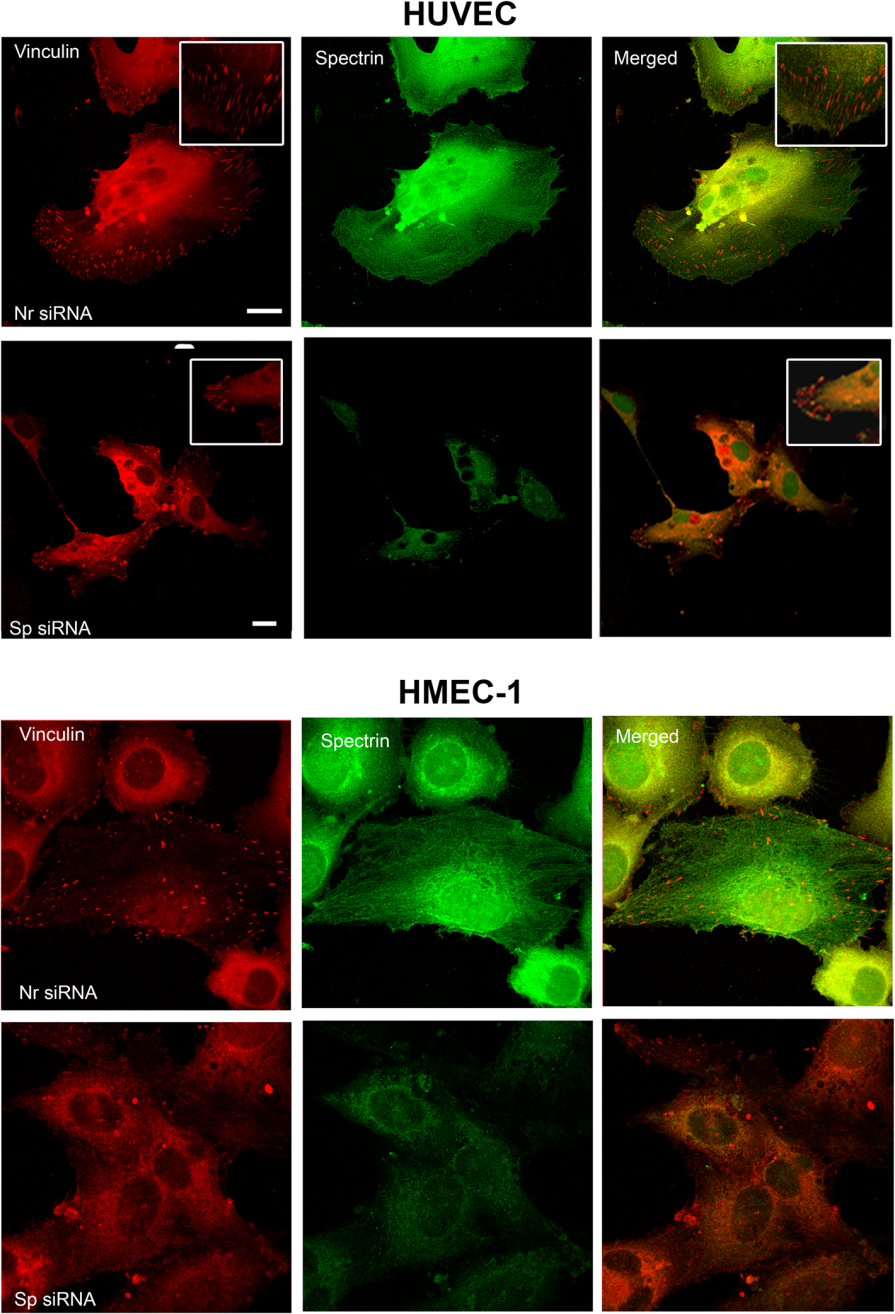
Spectrin depletion modifies focal points in endothelial cells. Focal points were analyzed using monoclonal antibodies directed against vinculin (red) and polyclonal antibodies directed against αII-spectrin (green) on HMEC-1 cells and HUVECs 72 h after transfection with Sp siRNA or Nr siRNA. Spectrin-depleted cells exhibited a reduced number of focal points compared to Nr siRNA-treated cells. Scale bar = 20 μm

As spectrin-depleted cells exhibit adhesion and spreading defects, the adhesion structures such as focal points were analyzed using antibodies directed against vinculin, a component of these structures (Fig. 4). Spectrin-depleted HMEC-1 cells and HUVECs presented fewer focal points.

**Fig. 5:**
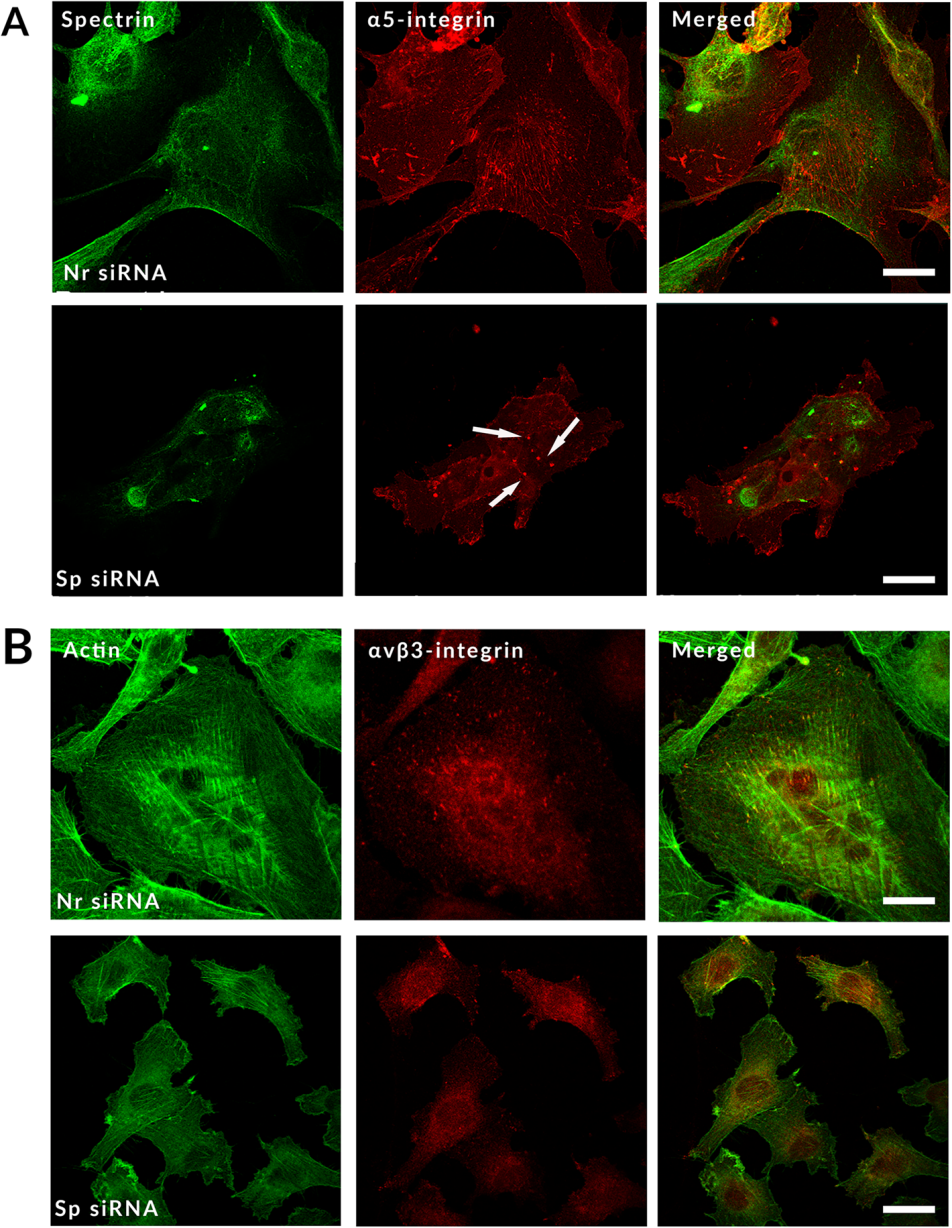
Knockdown of αII-spectrin alters the expression and localization of some integrins. **a** and **b** HMEC-1 cells were labelled 72 h after transfection with either Nr siRNA or Sp siRNA with polyclonal antibodies (red) directed against α5-integrin (**a**) and αVβ3-integrin (**b**). Spectrin and actin were labelled green. In Sp siRNA-treated cells, α5-integrin distribution is modified (present as aggregates) and αVβ3-integrin does not accumulate in focal points and forms aggregates in the cytoplasm. Depletion of spectrin is also associated with decreased expression of these integrins. Scale bar = 20 μm

Concerning adhesion molecules, we observed a modified location for α5-integrin and αVβ3-integrin. Depletion of spectrin is associated with aggregates of α5-integrin. We also observed the absence of αVβ3-integrin accumulation at the focal points. Instead, it formed aggregates in the cytoplasm, most of them without actin labelling (Fig. 5). As shown above, spectrin-depleted cells present a reduced size. Therefore, knockdown of αII-spectrin is associated with a modification of adhesion structures and an abnormal distribution of the main adhesion proteins.

### Spectrin depletion affects the formation of capillary tube structures on Matrigel

Since spectrin loss alters endothelial cell adhesion and spreading, we investigated the involvement of αII-spectrin in the physiological mechanism of angiogenesis based on these two processes. Endothelial cells plated on Matrigel reorganize and form characteristic structures called capillary tube structures in a process that mimics angiogenesis. The ability of spectrin-depleted cells to participate in the formation of capillary tube structures on Matrigel was tested.

**Fig. 6:**
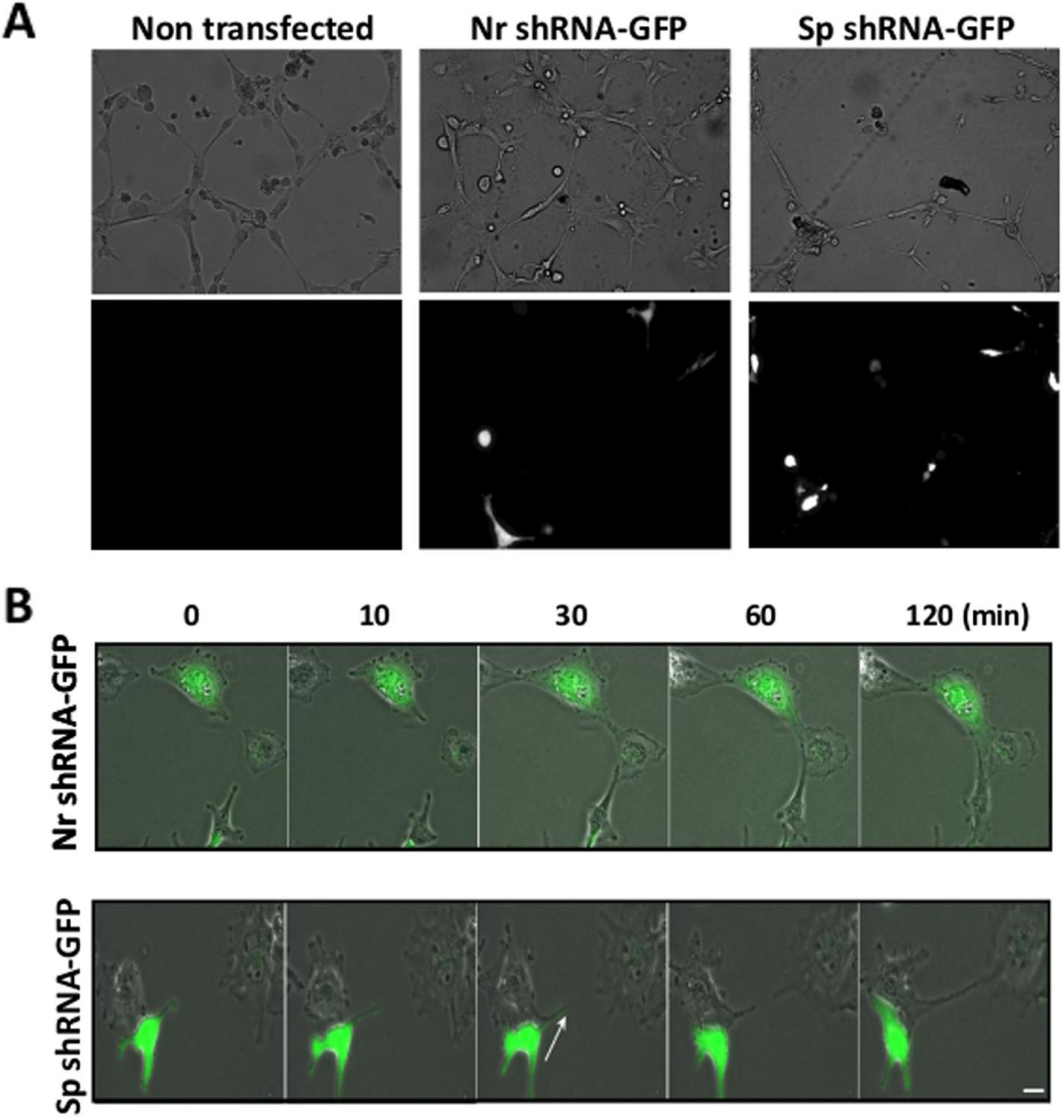
Knockdown of αII-spectrin disturbs capillary tube formation in vitro. **a** Observation of capillary tube formation on Matrigel. HMEC-1 cells transfected with plasmids coding for GFP and either Nr shRNA or Sp shRNA were seeded 72 h after transfection at a density of 100,000 cells/ml on Matrigel (9.3 mg/ml) and observed 10 h after plating. Capillary tube formation is not disturbed in cells treated with Nr shRNA (middle panel) compared to the formation by non-transfected cells (left panel). The presence of spectrin-depleted cells (right panel) alters the formation of these structures in vitro: capillary tubes are less dense and remain incomplete. Camera, 10x magnification. Lower panel – fluorescent images of HMEC-1 cells transfected with plasmid coding GFP. **b** Kinetics of capillary tube formation. HMEC-1 cells transfected for 72 h with plasmids coding for GFP and either Nr shRNA or Sp shRNA were seeded on Matrigel and observed using video microscopy for 12 h. Spectrin-depleted cells form extensions but these extensions appear labile and disappear very quickly (arrow) as compared to cells transfected with control shRNA. Scale bar = 20 μm

These experiments were performed on HMEC-1 cells transfected with a plasmid coding for both shRNAs and GFP. 72 h after transfection, depletion of αII-spectrin was analyzed via western blot and found to be efficient: its residual expression was around 35%. Cells transfected with non-relevant shRNA (Nr shRNA-GFP) show similar capillary tube formation to non-transfected cells (Fig. 6a), with most of the transfected cells participating in capillary tube formation. By contrast, the presence of spectrin-depleted cells (Sp shRNA-GFP) disturbs the capillary tube formation and it appears that most of these cells do not participate in the formation of the network. The Sp shRNA-GFP capillary tube network is less dense and remained incomplete at the end of the process (Fig. 6a). Spectrin-depleted cells were not completely spread as compared to non-transfected cells and cells transfected with a non-relevant shRNA that provides long extensions (filopodia).

**Fig. 7:**
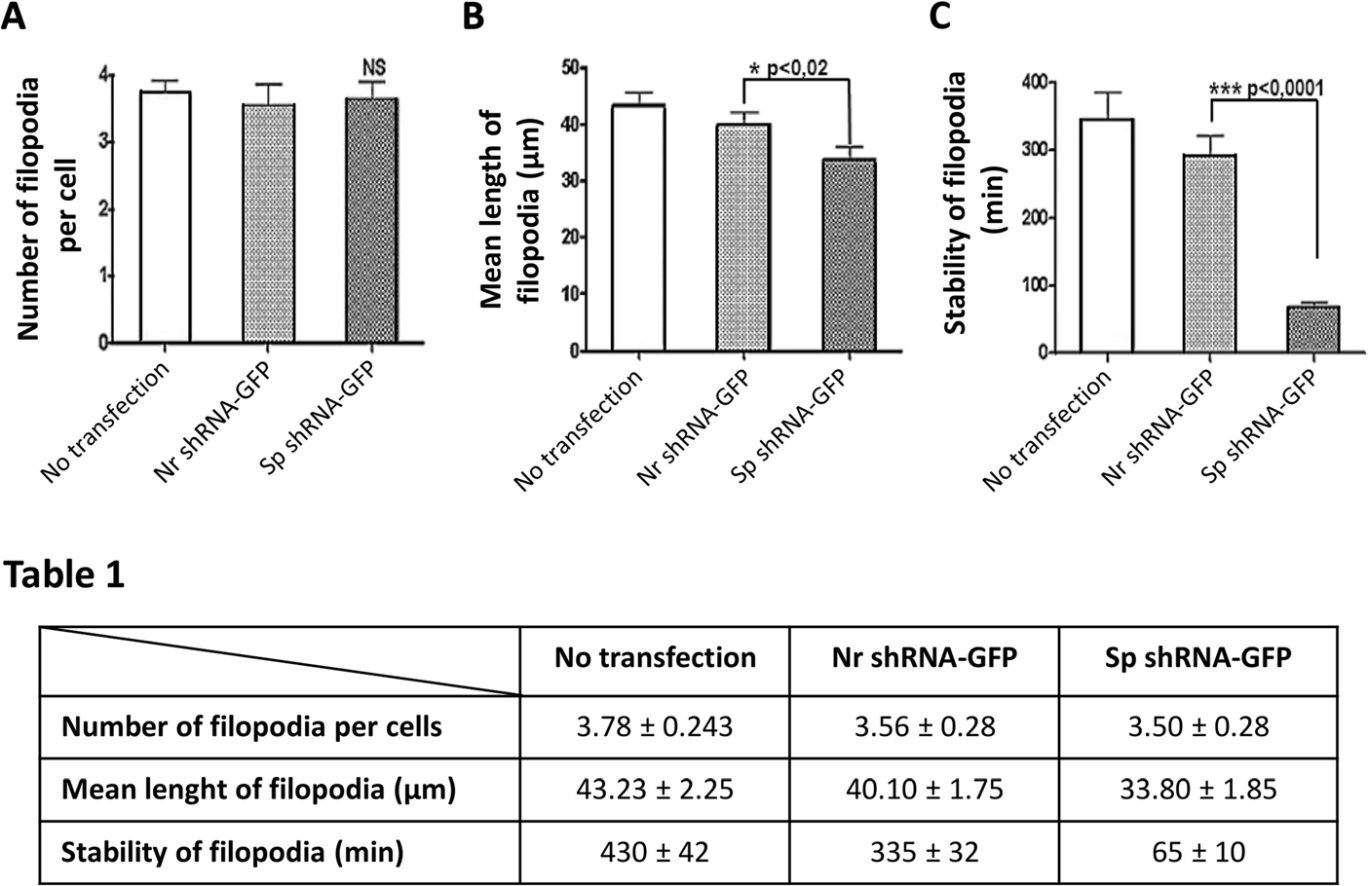
Knockdown of αII-spectrin destabilizes cell projection. **a** through **c** The number of cell extensions (filopodias) and their maximum size and lifetime (stability) were evaluated in HMEC-1 cells which were transfected or not with plasmids expressing GFP and Nr shRNA or Sp shRNA. The number of filopodia per cell is not significantly affected in cells transfected with shRNA targeting αII-spectrin (**a**; Bartlett’s test ANOVA, *p* = 0.8487), but their length is significantly reduced (**b**; Bartlett’s test, *p* = 0.0076) and their lifespan is dramatically decreased (**c**; Mann Whitney test, *p* = 0.0006)

Video microscopy analysis during capillary tube formation revealed that spectrin-depleted cells form extensions and initiate contact. However, these extensions and contacts do not seem stable as the filopodia retract. As a result, spectrin-depleted cells are excluded from the capillary tube (Fig. 6b, arrows). The number of filopodia per cell was similar to that observed in Nr shRNA-GFP treated cells and was around 3.5 vs 3.7 in non-transfected cells (Fig. 7a). However, the spectrin-depleted cell filopodia are shorter than those of control cells or non-relevant transfected shRNA cells (43.23 ± 2.25 in non-transfected cells, 40.10 ± 1.75 in Nr shRNA-GFP-transfected cells and 33.8 ± 1.85 in Sp shRNA-GFP-transfected cells; Fig. 7b). The most relevant feature is the shorter stability of extensions in spectrin-depleted cells compared to the control cells and Nr shRNA-GFP-transfected cells (stable for 430 ± 42 min in control cells, 335 ± 32 min in Nr shRNA-GFP-transfected cells and 85 ± 10 min in Sp shRNA-GFP-transfected cells; Fig. 7c). Spectrin-depleted cells could emit extensions, but the lifespan of these extensions is around six times shorter than those observed in control and non-relevant shRNA transfected cells. Depletion of αII-spectrin alters the stability of cellular extensions, which could participate in the defective formation of the capillary tube.

**Fig. 8:**
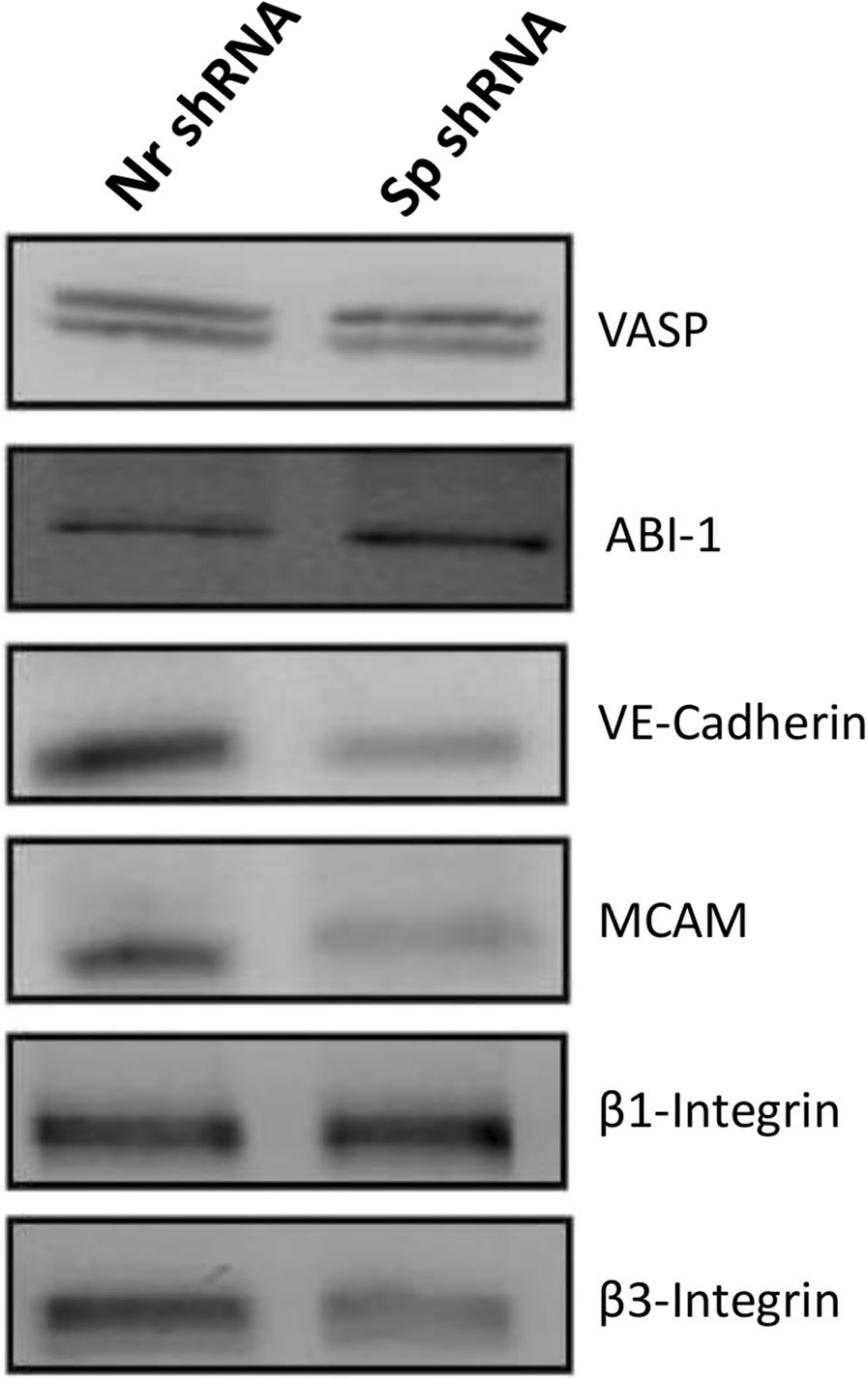
αII-spectrin depletion is associated with reduced expression of proteins involved in capillary tube assembly and formation. HMEC-1 cells were transfected with plasmids expressing either non-relevant shRNA (Nr shRNA) or shRNA directed against αII-spectrin (Sp shRNA). Western blot analyses were performed 72 h after transfection on cell lysates (20 μg). Spectrin depletion does not disturb expression of VASP, ABI-1 or β1-integrin. The expressions of VE-Cadherin, MCAM and β3-integrin were significantly reduced in cells transfected with Sp shRNA

Recent findings show that αII-spectrin is a partner of two proteins, VASP and Abi1, which are involved in cell–cell contacts [16, 17]. In particular, spectrin participates in the recruitment of VASP at the cell membrane and αII-spectrin–VASP complexes regulate the cytoskeleton assembly at endothelial cell–cell contacts. Therefore, we assessed VASP and ABI-1 expression. Western blot did not reveal any differences in the expression of these proteins in spectrin-depleted cells. We also analyzed the expressions of adhesion proteins involved in the formation and/or assembly of the capillary tube: VE-cadherin, MCAM, and β1- and β3-integrins. Western blot revealed a considerable decrease in the expression of VE-cadherin (80%), MCAM (75%) and β3-integrin (70%), but β1-integrin expression was not changed (Fig. 8).

## Discussion

Many recent reports have highlighted the importance of spectrin in maintaining cell shape, physical properties and membrane integrity and controlling the localization and stability of many surface proteins. The absence of homologous αII-spectrin in *D. melanogaster*, *C. elegans* and mice is associated with abnormal development and lethality [10, 18–20].

Organ development and size can be stimulated by various factors. Biophysical mediators can induce changes in the actin dynamic to elicit gene-specific transcription [21]. Recent findings strongly indicate that the spectrin network is essential for cell spreading, tissue differentiation and growth of organs, but little is known about its development-related roles in vertebrates. In this study, we focused on the functions of spectrin in endothelial cells and its role in the control of morphogenesis.

### Spectrin regulates the organization of actin skeleton

We previously reported that αII-spectrin depletion is associated with modifications of the actin skeleton, mainly characterized by the disappearance of stress fibers in a melanoma cell line [12] and the actin-rich lamellipodia in T cells [15]. These observations were validated in embryonic fibroblasts from an spna2^−^/^−^ mouse model that also presented the loss of stress fibers and the absence of cortical actin in lamellipodia [10]. Moreover, reducing the expression of αII-spectrin in endothelial cells affects the organization of actin-rich invadosomes and reduces their ability to invade [14]. In neurites with reduced expression of αII-spectrin, cortical F-actin loss and changes in shape are observed [13]. In endothelial cells, we confirmed the disturbance of actin organization, associated again with a loss of actin stress fibers. Epithelial cells overexpressing β-spectrin had a changed morphology/changed their morphology and were degraded [22]. However, the SH3 domain of αII-spectrin has been revealed to bind proteins involved in actin dynamics, such as Abi1, EVL, VASP and Tes [16, 17, 23, 24]. Abi1 (also called e3b1), which is part of the WAVE protein complex, is involved in reorganization of the actin cytoskeleton: the direct interaction of Abi1 with WAVE2 increases WAVE2 activity for actin polymerization [25]. Two members of the Mena/VASP family, EVL and VASP, are situated in focal adhesions and filopodias [16]. Moreover, αII-spectrin–VASP complexes were reported to regulate cytoskeleton assembly at endothelial cell–cell contacts [23]. Rotter et al. [24] highlight a direct interaction between αII-spectrin and Tes, which is a tumor suppressor found along the actin stress fibers and at focal adhesion points. Tes interacts with vinculin, zyxin, talin or EVL, Mena and VASP, which are various proteins of cytoskeleton focal adhesion [26, 27]. The depletion of Tes in cells leads to damage of the actin stress fibers [27]. Furthermore, it was reported that αII-spectrin participates in the Rac activation for actin filament formation and spreading through its SH3 domain [28]. We also hypothesized that spectrin, through direct interaction with VASP, indirectly controls the activation of talin, thus participating in the regulation of LFA1 integrin clustering in the immunological synapse region [15]. The possible integration between the spectrin-based skeleton and the proteins involved in actin dynamics suggests a new way of linking the spectrin-based skeleton with actin reorganization.

### Spectrins play a role in the control of cell adhesion and migration

Using siRNA approaches, we previously found that αII-spectrin, which is constitutively expressed in all nucleated cells, is also an main actor for nucleated cell shape and cell–matrix adhesion [12–15]. As this study showed, depletion of αII-spectrin in endothelial cell lines is associated with defective adhesion, loss of cell spreading, modification of cell shape, and fewer, less dense and less regular focal adhesion points. Similarly, siRNA-mediated depletion of βII-spectrin in epithelial cells modifies the cell shape with a loss of the lateral membrane [29]. Other studies revealed that other elements of the spectrin-based skeleton, such as ankyrin-G, are involved in interactions with βII-spectrin related to epithelial cell polarity and the formation of the lateral membrane [30]. As shown recently, protein 4.1R is also implicated in adhesion, spreading, migration and motility of mouse keratinocytes [31, 32].

In a melanoma cell line [12], we observed that depletion of αII-spectrin in endothelial cells alters cell adhesion and *a fortiori* spreading. The defect is more pronounced on laminin (the β3-integrin ligand) than on fibronectin (the β1-integrin ligand). These changes are associated with decreased expression of β1-integrin, which remains normally distributed, and with accumulation aggregates of αVβ3-integrin which remains normally expressed. Furthermore, αII-spectrin depletion induces a decreased number of focal adhesions points associated with modification of their architecture. Therefore, decreased expression of αII-spectrin may lead to inefficient accumulation of integrins in the membrane and disorganization of focal adhesion points.

αII-spectrin accumulates in specialized integrin clusters that initiate cell adhesion [28]. The SH3 domain of αII-spectrin colocalizes with β3-integrin at adhesion sites and disappears at a later stage of cell spreading. On the other hand, recent data indicates extensive regulation of integrins by Abi1 [33]. Spectrin downregulation, which affects cell–matrix adhesion and impairs spreading, can be associated with Abi1, which is directly bound to the spectrin SH3 domain [17]. This connection may be regulated by phosphorylation of Abi1, not only by the levels of this protein.

Our results highlight an essential role of non-erythroid spectrin in cell adhesion, regardless of the cell context. They also confirm the link between spectrin and adhesion molecules such as β3-integrin. Spectrin, in both RBCs and non-erythroid cells, is found in adhesion complexes involved in cell adhesion to the extracellular matrix and in regulation of cell–cell contact. Details of these specific interactions are presented in a new review [9]. Numerous studies have shown that immunoglobulin superfamily cell adhesion molecules (CAMs) can regulate the cytoskeleton and that the cytoskeleton directly controls the function and level of CAMs, for example in neurons [34].

We also observed decreased expression of the two endothelial adhesion molecules, VE-cadherin and MCAM, in spectrin-depleted cells. This decreased expression of adhesion molecules may be the reason for the observed defect in adhesion with the extracellular matrix. As yet, there are no data confirming a link between αII-spectrin and these molecules.

### Spectrin is engaged in angiogenesis

Angiogenesis is a physiological mechanism based on cell adhesion and spreading. We revealed for the first time that spectrin may be involved in this process. We observed that αII-spectrin depletion impairs capillary tube formation in vitro*.* Spectrin depletion (knockdown of αII-spectrin) disturbs the stability of cell projections and cell–cell contacts, but also correlates with a decreased level of VE-cadherin, MCAM and β3-integrins, three proteins involved in angiogenesis [35]. MCAM shows pro-angiogenic potential: it is a membrane signal receptor in tumor-induced angiogenesis [36] and has been identified as a novel target for anti-angiogenic agents in anticancer therapy [37]. Moreover, αvβ3 integrin, which is upregulated in tumor-associated blood vessels, has received much attention because of its anti-angiogenic potential [38].

In conclusion, αII-spectrin seems to be involved in the expression of proteins strongly engaged in angiogenesis under both physiological and pathological conditions. We conclude that non-erythroid spectrin may play a crucial role in the control of endothelial cell–matrix contact and migration, and that its depletion leads to impairment of angiogenesis in vivo*.*

## Supplementary information

**Additional file 1: Figure S1.** The distribution of αII-spectrin in endothelial cells: HMEC-1 cells and HUVECs were labeled with polyclonal antibodies directed against αII-spectrin. Scale bar = 20 μm.

**Additional file 2: Figure S2.** αII-spectrin depletion modified actin dynamics in living HUVECs. HUVECs expressing GFP-actin and transfected with siRNAs (Sp and Nr siRNAs) were analyzed using video microscopy 72 h after transfection. Cells treated with Sp siRNAs exhibit a reduced size and global disorganization of the actin network. Actin mainly accumulates in dynamic patches when in cells treated with Nr siRNAs. Actin formed very dynamic stress fibers and accumulated at the leading edge of the cells.

**Additional file 3: Figure S3A.** Video illustrating the Nr siRNA case for Fig. 2c.

**Additional file 4: Figure S3B.** Video illustrating the Sp siRNA case for Fig. 2c.

**Additional file 5: Figure S4.** Raw image file for western blots shown in Fig. 1a.

## Data Availability

The datasets used and/or analyzed during this study are available from the corresponding author upon reasonable request.

## Abbreviations

Abi1: Abelson interactor 1
CAMs: Cell adhesion molecules
EC: Endothelial cell
EVL: Ena/VASP-like protein
GFP: Green fluorescence protein
HMEC-1: Human microvascular endothelial cell
HUVECs: Human umbilical vein endothelial cells
IS: Immunological synapse
LFA1: Lymphocyte function-associated antigen 1
MCAM: Melanoma cell adhesion molecule
RBC: Red blood cell
SH3 domain: SRC Homology 3 Domain
shRNA: Short hairpin RNA
siRNA: Small interfering RNA
VASP: Vasodilator-stimulated phosphoprotein
VE-cadherin: Vascular endothelial cadherin
