# Supplementary figures and images for: Deficiency of αII-spectrin affects endothelial cell–matrix contact and migration leading to impairment of angiogenesis in vitro
Source: Cell Mol Biol Lett. 2020 Feb 3;25:3. doi: 10.1186/s11658-020-0200-y (PMC6998227; doi:10.1186/s11658-020-0200-y)

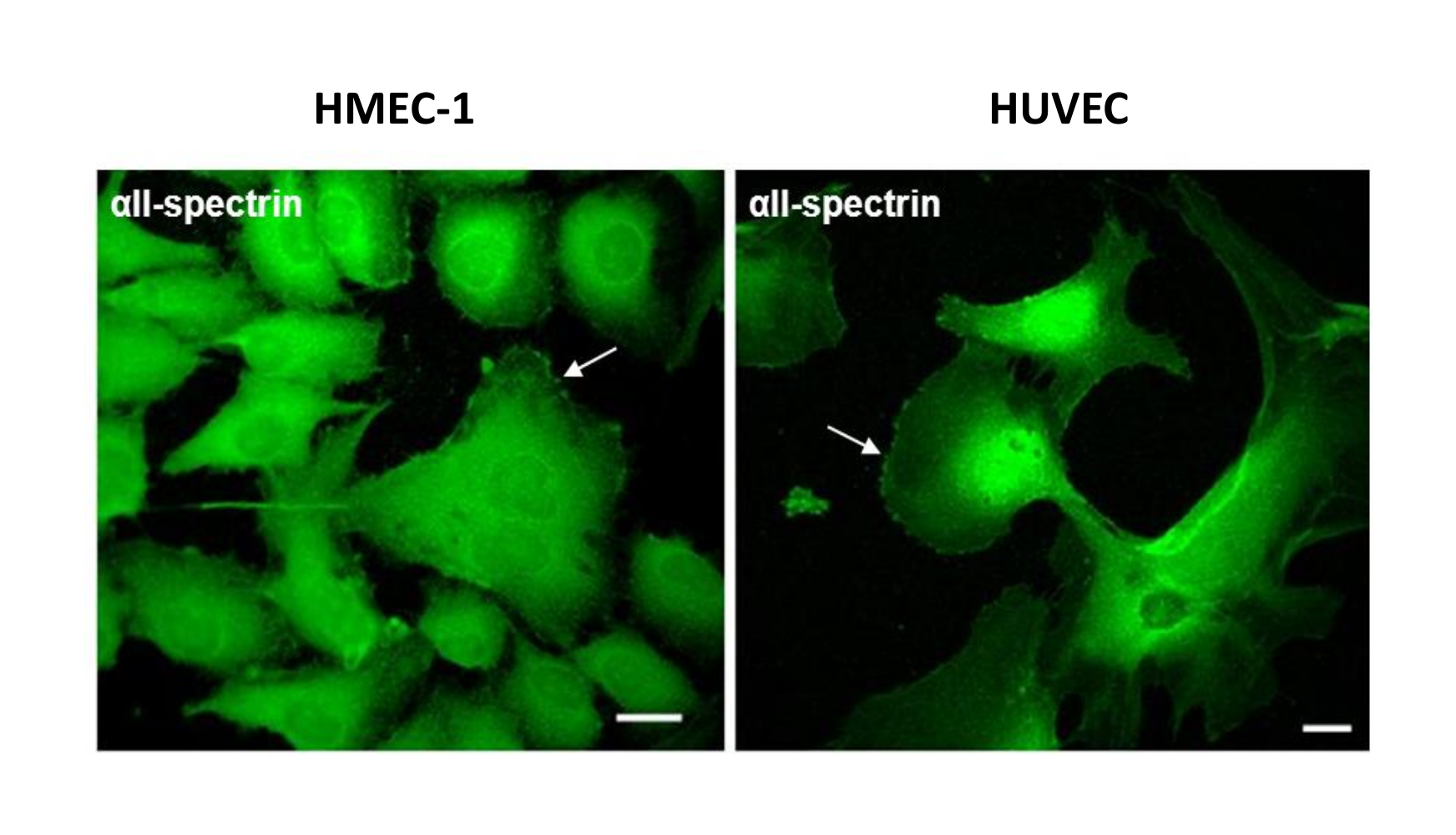

Supplement: Supplementary file 1 — Additional file 1: Figure S1. The distribution of αII-spectrin in endothelial cells: HMEC-1 cells and HUVECs were labeled with polyclonal antibodies directed against αII-spectrin. Scale bar = 20 μm. [file 11658_2020_200_MOESM1_ESM.tiff]

HUVEC HMEC-1

sp siRNA Nr siRNA sp siRNA Nr siRNA

αII spectrin


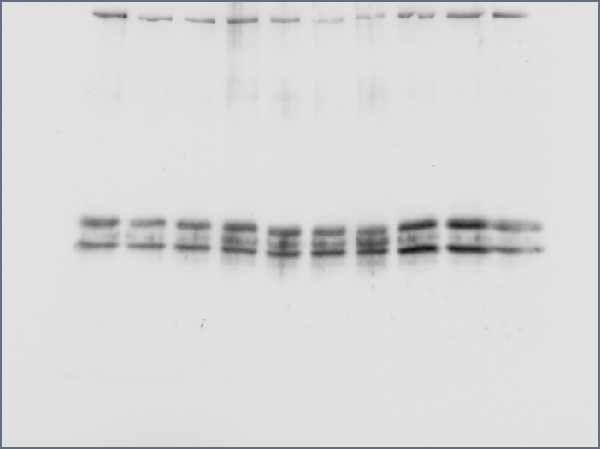

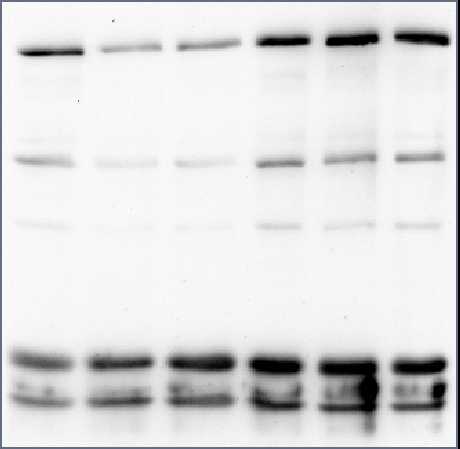


Lamin A/C

Supplement: Supplementary file 5 — Additional file 5: Figure S4. Raw image file for western blots shown in Fig. 1a. [file 11658_2020_200_MOESM5_ESM.docx]
